# Scientific production on instruments adapted to native languages in the Peruvian context

**DOI:** 10.3389/fpsyg.2025.1628131

**Published:** 2025-11-25

**Authors:** Ruth M. Phoco-Arhuiri, Joel Figueroa-Quiñones, Julio Cjuno

**Affiliations:** 1 Graduate School of Psychology, Universidad Peruana Unión, Lima, Peru; 2 School of Psychology, Universidad Autonoma de Ica, Ica, Peru

**Keywords:** psychometric properties, indigenous peoples, Peru, validation study, Quechua speaking

## Abstract

**Objective:**

To describe the scientific output on instruments adapted to Indigenous languages in Peru.

**Methods:**

A narrative review was conducted based on a search in Scopus, SciELO, PubMed, and LILACS using search terms related to psychometric properties, Peru, and Indigenous languages, refining the search by title, abstract, and keywords. The search was conducted in January 2025, yielding 9 studies in Scopus, 4 in SciELO, 28 in PubMed, and 10 in LILACS. After removing duplicates and excluding studies that did not meet the inclusion criteria, a final sample of *n* = 6 studies was obtained.

**Results:**

The instruments adapted to certain Quechua varieties assessed depression, anxiety, general mental health, overall well-being, and life satisfaction. Five out of six studies were conducted with bilingual adult populations. A smaller number of items corresponded to smaller sample sizes (e.g., *n* = 186 as the minimum for 5 items). The studies examined sources of validity including internal structure, content, criterion-related evidence, and measurement invariance. For reliability, they used classical Alpha coefficients and McDonald’s Omega.

**Conclusion:**

Few studies were found on the adaptation of psychometric instruments to certain Quechua varieties. The instruments addressed the assessment of depression, anxiety, general well-being, life satisfaction, and overall mental health. University authorities in Peru could foster psychometric research initiatives within Indigenous communities.

## 1 Introduction

Globally, there are approximately 7,000 languages, of which around 4,000 are Indigenous languages spoken by about 370 million people, representing 6% of the world’s population (UNESCO, 2019). In Latin America, the highest proportion of Indigenous populations is found, with over 42 million people residing in the region, which accounts for 11% of the global Indigenous population (Banco Mundial, 2015). In fact, the countries with the highest number of Indigenous languages are Brazil, with 186 languages; Mexico, with 67; Colombia, with 65; Peru, with 55; and Venezuela, with 37 (Banco Mundial, 2015).

Through the native language of Indigenous populations, their identity and culture are transmitted, along with their traditions, knowledge, values, and emotions, all of which enrich global cultural diversity (Semali and Kincheloe, 1999). For this reason, international organizations have emphasized that the preservation and promotion of Indigenous languages is not only a cultural right but also a means to strengthen sustainable development and social inclusion (UNESCO, 2019). Indeed, studies involving these populations present methodological and ethical challenges in assessment, since the use of tests designed in these languages requires careful procedures to reduce biases that may affect the validity of results (Gone and Kirmayer, 2020).

Scientific production aimed at the adaptation of instruments in native languages is limited, despite the widespread use of such languages, especially in Latin America (Cjuno et al., 2021). The adaptation of psychological tests to different cultural contexts has been widely discussed, highlighting the importance of rigorous processes that consider not only linguistic translation, but also semantic, conceptual, and metric equivalence (Hambleton et al., 2004). Likewise, in the case of Indigenous languages, these processes face additional challenges, such as the scarcity of bilingual experts, the lack of technical terminology in some languages, and differences in the cultural reference frameworks of the communities (Leong et al., 2016).

Peru is a country characterized by its rich cultural and linguistic diversity, with 55 officially recognized native languages, 51 of which are spoken in the Amazon region and 4 in the Andean region (Ministerio de Educación, 2017). Some studies have revealed that the majority of research on the adaptation or validation of mental health screening instruments in Peru focuses on university students or adults, while studies involving native languages remain a pressing need (Cjuno et al., 2022). Other authors emphasize the importance of incorporating concepts and terms specific to Indigenous languages to ensure the comprehension and acceptance of instruments by the communities (Sillocca, 2020). Similarly, some studies stress that the active participation of community members in the adaptation process helps strengthen the ecological validity of such studies (Santiago, 2025). This scenario poses challenges for scientific research, particularly in the creation and adaptation of instruments that are culturally and linguistically appropriate for these communities. The absence of validated tools in Indigenous languages is a limiting factor for both the inclusion of Indigenous populations in scientific studies and the generation of reliable data for designing public policies in their favor (Saucier and Goldberg, 2001; Mamani-Benito et al., 2023).

Therefore, the present narrative review study aims to describe the current state of scientific production on instruments adapted to Indigenous languages in Peru, identifying progress, limitations, and future perspectives. By addressing this topic, the study seeks not only to contribute to the visibility of these initiatives but also to promote a more inclusive research agenda that represents the country’s cultural diversity.

## 2 Materials and methods

The present study is a narrative review aimed at descriptively evaluating and synthesizing the results of studies on instruments adapted to Indigenous languages in the Peruvian context. This approach is justified by the variety of research designs involved, which allow for the integration of theories from different approaches and perspectives, providing a more cohesive understanding of the behavior of these study variables (Ato et al., 2013).

### 2.1 Information sources

To conduct this review, searches of the scientific literature were carried out up to January 2025 across four databases: Scopus, SciELO, PubMed, and LILACS, considering studies published in English and/or Spanish. The search strategy was developed using terms and descriptors related to scientific production on instruments adapted to Indigenous languages in the Peruvian context. In PubMed, field tags were used to search titles and abstracts; in SciELO and LILACS, all fields were used; and in Scopus, searches were conducted in titles and abs-key. This strategy is detailed in Supplementary Appendix 1.

### 2.2 Eligibility criteria

Once the search strategy was finalized, the researcher proceeded to search the databases mentioned above. The results were organized using Microsoft Excel. Included in the review were studies on instruments adapted to Indigenous languages in the Peruvian context, such as original articles, short original articles, and systematic reviews.

### 2.3 Study selection process

Excluded from the review were items such as notes and errata, duplicate entries, studies not conducted in Indigenous languages, and studies whose focus was unrelated to psychometric properties. Titles and abstracts were compared against inclusion and exclusion criteria. Based on the selected articles, the researcher reviewed and categorized the studies (included vs. excluded).

### 2.4 Data extraction process

From the included studies, the full text of each article was reviewed, and relevant data were extracted using a data collection form created in Microsoft Excel. The following variables were considered: first author and year of publication, main objective, sample characteristics, evidence of internal structure validity, reliability, external validity, and measurement invariance.

### 2.5 Effect measures

The measures considered included the values of KMO and Bartlett’s test, variance explained (in percentages), goodness-of-fit indices, reliability coefficients, CFI and RMSEA differences for invariance testing, and correlation coefficients for convergent validity.

**Figure 1:**
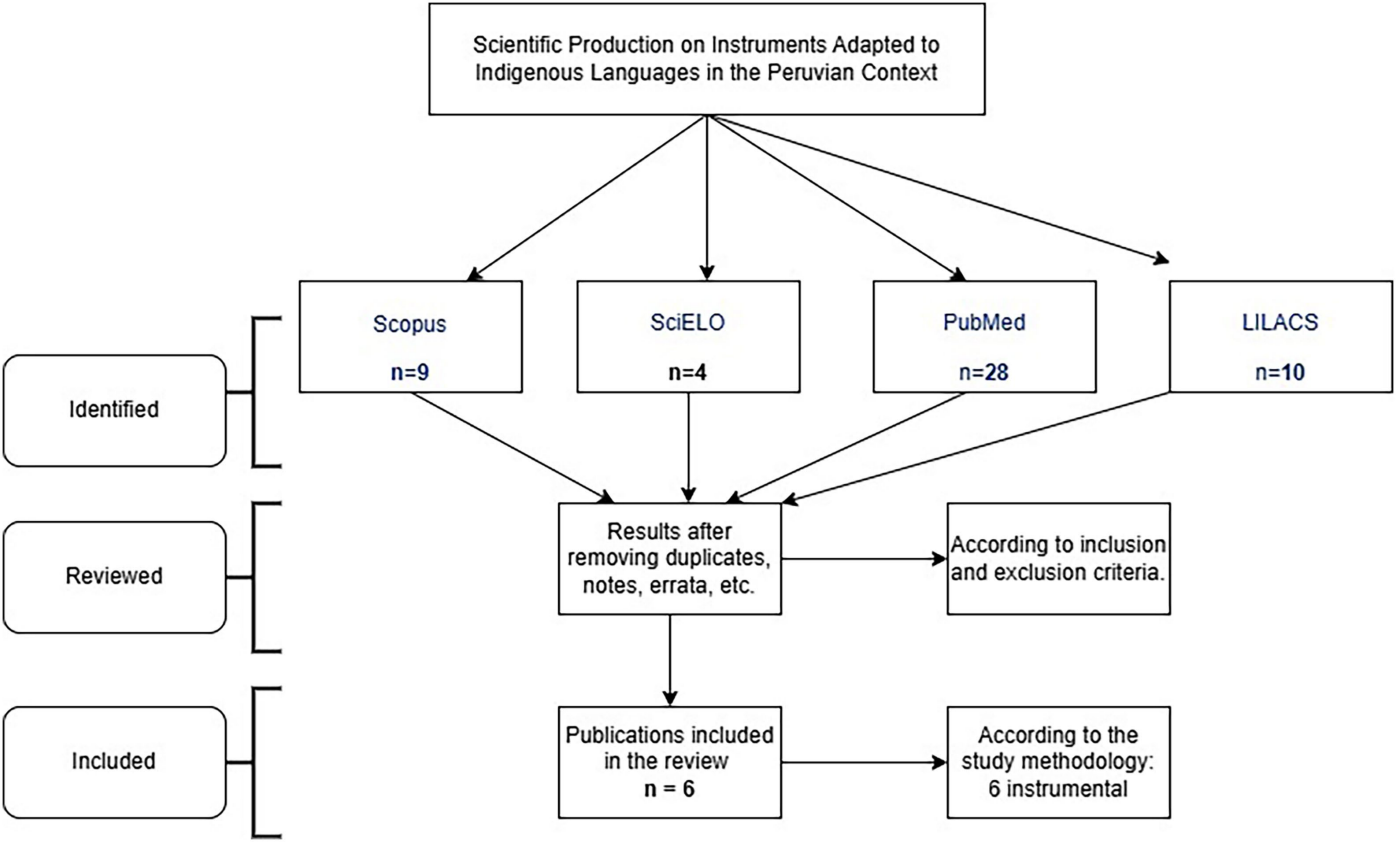
Flowchart of the study selection process.

Finally, all processes and stages of the study selection are presented in the following flowchart (Figure 1).

## 3 Results

Based on the search conducted in January 2025, *n* = 9 studies were found in Scopus, *n* = 4 in Scielo, *n* = 28 in PubMed, and *n* = 10 in Lilacs. After removing duplicates and studies that did not meet the inclusion criteria, we obtained a sample of *n* = 6 studies that sought to adapt instruments to an indigenous language of the Peruvian population (Figure 1).

We found that 5 out of 6 studies were conducted in recent years (2023 and 2024) (Mamani-Benito et al., 2023; Cjuno et al., 2023; Carranza Esteban et al., 2023; Carranza et al., 2024; Cjuno et al., 2024), 5 out of 6 studies were led by Peruvian researchers (Mamani-Benito et al., 2023; Cjuno et al., 2023; Carranza Esteban et al., 2023; Carranza et al., 2024; Cjuno et al., 2024), of which 3 out of 5 declared their city of residence as Lima (Carranza Esteban et al., 2023; Carranza et al., 2024; Cjuno et al., 2024). Additionally, 5 out of 6 corresponding authors were psychologists (Mamani-Benito et al., 2023; Cjuno et al., 2023; Carranza Esteban et al., 2023; Carranza et al., 2024; Cjuno et al., 2024), and 2 out of 6 listed the Universidad Peruana Unión as their primary affiliation, along with Universidad San Ignacio de Loyola (Mamani-Benito et al., 2023; Carranza Esteban et al., 2023; Carranza et al., 2024; Cjuno et al., 2024; Table 1).

**Table 1:** Characteristics of the corresponding author of the included studies.

First author (year)	Affiliation of corresponding author	Country and city of corresponding author	Profession of corresponding author
Tremblay et al. (2009)	McGill University	Canadá, Montreal	Physician
Mamani-Benito et al. (2023)	University Peruana Unión	Perú, Juliaca	Psychologist
Cjuno et al. (2023)	University Cesar Vallejo	Perú, Piura	Psychologist
Carranza Esteban et al. (2023)	University San Ignacio de Loyola	Perú, Lima	Psychologist
Esteban et al. (2024)	University San Ignacio de Loyola	Perú, Lima	Psychologist
Cjuno et al. (2024)	University Peruana Unión	Perú, Lima	Psychologist

This table presents the characteristics of the corresponding authors for the studies included in the review.

On the other hand, the adaptations of instruments were linked to health-related variables such as depression, anxiety, general mental health, overall well-being, and life satisfaction. All test adaptation studies were conducted into Quechua; of these, 5 out of 6 studies were carried out in Collao Quechua, also known as Cuzco-Collao (Mamani-Benito et al., 2023; Cjuno et al., 2023; Carranza Esteban et al., 2023; Carranza et al., 2024; Cjuno et al., 2024), 2 out of 6 in Chanca Quechua (Tremblay et al., 2009; Cjuno et al., 2023), and 1 out of 6 in Central Quechua (Cjuno et al., 2023; Table 2).

**Table 2:** Evidence of validity and reliability of the selected instruments.

First author (year)	Adapted instrument	Original language	Variable of interest	Participants	Sources of validity	Sources of reliability
Tremblay et al. (2009)	General Health Questionnaire (GHQ-12)	Quechua Chanca	General mental health	General mental health	373 Quechua-speaking participants from Ayacucho aged 15 to over 50, both sexes	Content validity, internal structure validity, criterion validity
Mamani-Benito et al. (2023)	Satisfaction With Life Scale (SWLS)	Quechua Collao	Life satisfaction	Life satisfaction	242 Quechua speakers aged 18 and older from the city of Puno, both sexes	Content validity, internal structure validity, measurement invariance
Cjuno et al. (2023)	Patient Health Questionnaire (PHQ-9)	Quechua Chanca, Quechua Cusco-Collao, Quechua Central	Depression	Depression	970 Quechua speakers (*n* = 219 Central, *n* = 226 Chanca, *n* = 525 Cusco-Collao) aged up to 70, both sexes from Ayacucho, Ancash, Puno, and Cusco	Content validity, internal structure validity, measurement invariance
Carranza Esteban et al. (2023)	General Well-being Index (WHO-5)	Quechua Collao	General well-being	General well-being	186 Quechua speakers aged 18 to 65, both sexes from the city of Puno	Content validity, internal structure validity
Carranza et al. (2024)	Patient Health Questionnaire for Depression and Anxiety (PHQ-4)	Quechua Collao	Anxiety and depression symptoms	221 Quechua speakers aged 18 to 65, both sexes from the city of Puno	Content validity, internal structure validity	Cronbach’s Alpha, McDonald’s Omega
Cjuno et al. (2024)	Generalized Anxiety Questionnaire (GAD-7)	Quechua Cuzco-Collao	Anxiety	660 Quechua speakers aged 18 to 70, both sexes from Puno and Cusco	Content validity, internal structure validity, measurement invariance	Cronbach’s Alpha, McDonald’s Omega

The sample size used ranged from 186 participants (e.g., 5-item scale) to 970 participants (e.g., 9-item scale) (Tremblay et al., 2009; Mamani-Benito et al., 2023; Cjuno et al., 2023; Carranza Esteban et al., 2023; Carranza et al., 2024; Cjuno et al., 2024; Table 2).

Regarding sources of validity, all studies presented content validity and internal structure validity (Tremblay et al., 2009; Mamani-Benito et al., 2023; Cjuno et al., 2023; Carranza Esteban et al., 2023; Carranza et al., 2024; Cjuno et al., 2024); meanwhile, 3 out of 6 reported measurement invariance (Mamani-Benito et al., 2023; Cjuno et al., 2023; Cjuno et al., 2024), and 1 out of 6 reported criterion validity (Tremblay et al., 2009; Tables 3, 4).

**Table 3:** Psychometric results of the studies.

Authors	KMO/Bartlett	Explained variance (%)/Factors	Goodness-of-fit indices (CFI, TLI, RMSEA, SRMR)	Reliability (Cronbach’s *α*/McDonald’s *ω*)	Invariance (*Δ*CFI/ΔRMSEA)	Convergent validity
Tremblay et al. (2009)	N. A	51.40%	N. A	α = 0.81	N. A	PTSD-R y LID = *r* = 0.61**; PTSD-R y Ansiedad = *r* = 0.50**; PTSD-R y Depresión = *r* = 0.47**
Mamani-Benito et al. (2023)	N.A	Unidimensional	CFI = 0.995; TLI = N.A.; RMSEA = 0.023; SRMR = 0.034	*ω* = 0.65 (total); ω hombres = 0.68; ω mujeres = 0.67	(ΔCFI > 0.01; ΔRMSEA > 0.01)	N.A
Cjuno et al. (2023)	N.A	Unidimensional	CFI = 0.990; TLI = 0.987; RMSEA = 0.071; SRMR = 0.048	α = 0.895; ω = 0.861	Quechua (ΔCFI = 0.001–0.018; ΔRMSEA = 0.001–0.007); Sex (ΔCFI ≤ 0.008; ΔRMSEA ≤ 0.017); Resident (ΔCFI ≤ 0.003; ΔRMSEA ≤ 0.013); Age, Status marital and Education (ΔCFI, ΔTLI, ΔRMSEA < 0.01)	N.A
Carranza Esteban et al. (2023)	N.A	Unidimensional	CFI = 0.990; TLI = 0.980; RMSEA = 0.080; RMR = 0.020; GFI = 0.980	α = 0.88 (IC 95%: 0.84–0.90)	N.A	N.A
Carranza et al. (2024)	N.A	Unidimensional	RMSEA = 0.121 (IC 90%: 0.045–0.210); SRMR = 0.029; CFI = 0.99; TLI = 0.97	α = 0.86; ω = 0.81	N.A	N.A
Cjuno et al. (2024)	KMO = 0.88; p de Bartlett = 0.01	Unidimensional	CFI = 0.994; TLI = 0.991; RMSEA = 0.092; SRMR = 0.027	*α* = 0.896; ω = 0.894	Sex (ΔCFI = 0.002–0.003; ΔRMSEA = 0.002–0.017). Status marital (ΔCFI = 0.002–0.004; ΔRMSEA = 0.002–0.023); Age and Education level (ΔCFI, ΔTLI, ΔRMSEA < 0.01)	N.A

N.A, not applied; ω, omega coefficient; α, Cronbach’s alpha; Δ, delta; PTSD-R, Post-Traumatic Stress Disorder-Related; LID, Local Idioms of Distress; ** = Very significant.

**Table 4:** Content validity and cultural adaptation procedures.

Author(s)/year	Content validity method	Cultural adaptation procedure	Semantic equivalence	Conceptual equivalence	Aiken’s V index
Tremblay et al. (2009)	Expert judgment (translation reviewed by bilingual professionals)	Translation–back translation; committee review	Yes, confirmed by consensus of translators and experts	Yes, culturally relevant items were adjusted (e.g., symptoms expressed in context-specific terms)	N.A.
Mamani-Benito et al. (2023)	Expert judgment (3 psychologists with a master’s degree)	Direct translation by two translators, back translation, review by a committee, and focus group with native speakers	Yes, confirmed through the translation–back translation process, committee review, and focus group	Yes, the comprehension of the items and their adequacy to the Quechua cultural context were verified through the focus group	V > 0.70 in all items for relevance, representativeness, and clarity
Cjuno et al. (2023)	Expert judgment (6 specialists in psychometrics and mental health)	Direct translation, back translation, committee review, and cognitive interviews with native speakers	Yes, ensured through translation–back translation and pilot testing	Yes, the construct “depression” was culturally validated for Quechua (e.g., changes in expressions of sadness and somatization)	Aiken’s V > 0.70 in all items
Carranza Esteban et al. (2023)	Expert judgment (3 psychologists with a master’s degree)	Direct translation by two translators, back translation, committee review, and focus group with native speakers	Yes, confirmed through the translation–back translation process, committee review, and focus group. In addition, a critical analysis was conducted comparing with the original items in English	Yes, the comprehension and cultural adequacy of the items were verified through the focus group. A phrase was adapted to express “being in a good mood” in a culturally appropriate way (“imatapas ruway munaglla”).	> 0.70 in all items for relevance, representativeness, and clarity
Carranza et al. (2024)	Expert judgment (3 psychologists with a master’s degree and experience in bilingual treatment)	Direct translation by two translators, back translation, committee review, and focus group with native speakers.	Yes, confirmed through the translation–back translation process, committee review, and focus group	Yes, the comprehension and cultural adequacy of the items were verified through the focus group. No changes were suggested	V > 0.70 in all items for relevance, representativeness, and clarity
Cjuno et al. (2024)	Expert judgment (3 bilingual psychologists) using the Delphi method (2 rounds)	Direct translation, back translation, committee review, cultural adaptation through the Delphi method, and focus group	Yes, confirmed through the translation process and expert consensus. Specific terms were adapted (e.g., “anxiety” → “phutiskalla/ansiedad nisqhawan”)	Yes, the comprehension and cultural adequacy of the items and response categories were verified through the Delphi method and the focus group. “More than half the days” was adapted to “Ashka p’unchawkuna”	No numerical V values are reported, but a maximum score (3/3) is indicated for relevance, representativeness, clarity, and cultural equivalence for all items

N.A, not applied.

As for internal consistency, it was found that 3 out of 6 studies reported both Cronbach’s Alpha and McDonald’s Omega coefficients (Cjuno et al., 2023; Carranza et al., 2024; Cjuno et al., 2024), 2 out of 6 reported only Cronbach’s Alpha (Tremblay et al., 2009; Carranza Esteban et al., 2023), and 1 out of 6 reported only McDonald’s Omega (Mamani-Benito et al., 2023; Table 3).

## 4 Discussion

The instruments adapted to some varieties of the indigenous Quechua language assessed depression, anxiety, general mental health, overall well-being, and life satisfaction. A smaller number of items corresponded to a smaller sample size (e.g., n = 186 as the minimum for 5 items). The studies analyzed sources of validity such as internal structure, content, criterion-related validity, and measurement invariance; for reliability, they used both the classical Cronbach’s Alpha and McDonald’s Omega coefficients.

### 4.1 Benefited indigenous communities

Although studies were found that aimed to adapt psychological instruments to certain varieties of Quechua—such as Cuzco-Collao, Central, and Chanca—Quechua is a language family with diverse varieties across the Peruvian territory. These can be classified in two main ways: a generic classification including Cuzco-Collao, Chanca, Central, and the San Martín or jungle Quechua; and a more specific classification that divides them into multiple subgroups based on small variations from one Quechua-speaking community to another. This reflects that psychological instruments have not yet been fully adapted across the entire Quechua-speaking population.

Moreover, Peru is home to a diverse range of Indigenous peoples, totaling 55 distinct ethnic groups, each with its own language—51 located in the Amazon and 4 in the Andes. Therefore, the task of adapting instruments to each Indigenous community is still in its early stages.

### 4.2 Fields of knowledge addressed

While instruments adapted for screening mental health variables such as depression, anxiety, overall mental health, general well-being, and life satisfaction were found, the WHO has noted that Indigenous peoples are marginalized in their access to global health services (e.g., a lack of healthcare professionals who speak their language or culturally adapted tools). It is also known that they have a lower life expectancy compared to non-Indigenous populations and face higher rates of adverse health conditions such as diabetes, hypertension, maternal mortality, infant mortality, and chronic malnutrition (Muñoz-Del-Carpio-Toia et al., 2023; Garza and Abascal Miguel, 2025; Díaz et al., 2015). In light of this, it is evident that there is a pressing need to promote research teams focused on addressing the health and mental health of these communities, which often live in extreme poverty and lack access to dignified healthcare.

### 4.3 Can the instruments be used across the entire target population?

So far, the target population has been Quechua-speaking communities of Cuzco-Collao, Chanca, and Central varieties. However, we found that 5 out of 6 studies reported their samples were composed of bilingual Quechua speakers with a minimum level of education that allowed them to read in both Spanish and Quechua. This is likely due to the linguistic and methodological challenges of evaluating monolingual Quechua speakers (e.g., those who neither read nor write in Quechua). This is mainly because Quechua, as a language family, is traditionally spoken, and its speakers often have not received formal education, whereas bilingual Quechua speakers (e.g., those who can read and write in Spanish) are able to read and understand written Quechua.

Another reason for focusing on bilingual adults is that being bilingual does not necessarily change their beliefs, value systems, culture, or their unique way of understanding mental health issues. In this sense, being able to read and write in Spanish is not a sufficient reason to use a screening tool in Spanish in an Indigenous community with a culture, customs, beliefs, religious practices, and lifestyle different from those of Spanish-speaking populations. Linguistic competence alone is not enough; cultural particularities that influence the perception and management of mental health must also be taken into account.

One way to adapt a test for non-literate, monolingual Quechua speakers would be to develop an audio version of the test. Since Quechua is primarily spoken, listeners could understand each item and respond easily to an interviewer with limited Quechua training. However, developing audio-based instruments would require a larger budget (e.g., to collect and create a dataset of audio-recorded items and response options for training an AI model or hiring a software developer), as well as a previously adapted and validated written version of the instrument in the target Indigenous language. We believe that the editors and reviewers of the journals where 5 out of the 6 validation studies were published understood that studies with bilingual populations are a fundamental first step toward developing instruments that can eventually reach monolingual individuals.

### 4.4 Strengths and limitations

The strength of this study is that it is the first review to explore the scientific output on instruments adapted to Peruvian Indigenous languages. Its analysis provides insight into the progress made in addressing gaps in mental health and global health coverage in Indigenous communities. However, it does have some limitations, such as only including scientific literature indexed in Scopus, Web of Science, PubMed, Scielo, and Lilacs. It is possible that more studies exist in other databases such as PsycInfo. Nevertheless, since regional databases were included, we believe all relevant published studies to date were captured.

## 5 Conclusion

We found six studies on the adaptation of psychometric tests to some varieties of Quechua, which addressed the measurement of depression, anxiety, general well-being, life satisfaction, and overall mental health. These studies analyzed structural validity, criterion validity, content validity, measurement invariance, and reliability.

Peruvian universities and the authorities that oversee them could promote psychometric research initiatives in Indigenous communities to help bridge the significant gap in the availability of culturally adapted assessment tools across Peru.
