# Supplementary material for: Scientific production on instruments adapted to native languages in the Peruvian context
Source: Front Psychol. 2025 Nov 25;16:1628131. doi: 10.3389/fpsyg.2025.1628131 (PMC12685624; doi:10.3389/fpsyg.2025.1628131)
Supplement: Supplementary file 1 [file Supplementary_file_1.docx]

**Appendix 1**

**List of search strategies**

| **DATABASE** | **Search strategy** |
| --- | --- |
| SCOPUS | ( TITLE-ABS-KEY ( "psychometric properties" OR "validity" OR "reliability" OR "internal consistency" OR "factorial structure" OR "construct validity" OR "criterion validity" OR "test-retest reliability" OR "item analysis" OR "measurement properties" OR "measurement invariance" )  AND TITLE-ABS-KEY ( "Achuar" OR "Aimara" OR "Amahuaca" OR "Arabela" OR "Ashaninka" OR "Asheninka" OR "Awajún" OR "Bora" OR "Cashinahua" OR "Chamicuro" OR "Chapra" OR "Chitonahua" OR "Ese Eja" OR "Harakbut" OR "Ikitu" OR "Iñapari" OR "Iskonawa" OR "Jaqaru" OR "Jíbaro" OR "Kakataibo" OR "Kakinte" OR "Kandozi" OR "Kapanawa" OR "Kichwa" OR "Kukama Kukamiria" OR "Madija" OR "Maijuna" OR "Marinahua" OR "Mashco Piro" OR "Mastanahua" OR "Matsés" OR "Matsigenka" OR "Muniche" OR "Murui-Muinanɨ" OR "Nahua" OR "Nanti" OR "Nomatsigenga" OR "Ocaina" OR "Omagua" OR "Quechuas" OR "Resígaro" OR "Secoya" OR "Sharanahua" OR "Shawi" OR "Shipibo-Konibo" OR "Shiwilu" OR "Ticuna" OR "Urarina" OR "Uro" OR "Vacacocha" OR "Wampis" OR "Yagua" OR "Yaminahua" OR "Yanesha" OR "Yine" ) OR TITLE-ABS-KEY ( languages AND originating )  AND TITLE-ABS-KEY ( peruvians ) )  AND PUBYEAR > 2010 AND PUBYEAR < 2025 AND ( LIMIT-TO ( DOCTYPE , "ar" ) ) |

| **DATABASE** | **Search Query** |
| --- | --- |
| SCIELO | (("propiedades psicométricas" OR "validación" OR "confiabilidad" OR "consistencia interna" OR "estructura factorial" OR "validez de constructo" OR "validez de criterio" OR "confiabilidad test-retest" OR "análisis de ítems" OR "propiedades de medición " OR "invariancia de medición"))  AND (("Achuar" OR "Aymara" OR "Amahuaca" OR "Arabela" OR "Ashaninka" OR "Asheninka" OR "Awajún" OR "Bora" OR "Cashinahua" OR "Chamicuro" OR "Chapra" OR "Chitonahua" OR "Ese Eja" OR "Harakbut" OR "Ikitu" OR "Iñapari" OR "Iskonawa" OR "Jaqaru" OR "Jíbaro" OR "Kakataibo" OR "Kakinte" OR "Kandozi" OR "Kapanawa" OR "Kichwa" OR "Kukama Kukamiria" OR "Madija" OR "Maijuna" OR "Marinahua" OR "Mashco Piro" OR "Mastanahua" OR "Matsés" OR "Matsigenka" OR "Muniche" OR "Murui-Muinanɨ" OR "Nahua" OR "Nanti" OR "Nomatsigenga" OR "Ocaina" OR "Omagua" OR "Quechua" OR "Resígaro" OR "Secoya" OR "Sharanahua" OR "Shawi" OR "Shipibo-Konibo" OR "Shiwilu" OR "Ticuna" OR "Urarina" OR "Uro" OR "Vacacocha" OR "Wampis" OR "Yagua" OR "Yaminahua" OR "Yanesha" OR "Yine"))  AND ((peruano OR peru) OR AB=(peruano OR peru) OR (peruano OR Perú)) |

| **DATABASE** | **Search Query** |
| --- | --- |
| PUBMED | ("psychometric properties"[Title/Abstract] OR "validity"[Title/Abstract] OR "reliability"[Title/Abstract] OR "internal consistency"[Title/Abstract] OR "factorial structure"[Title/Abstract] OR "construct validity"[Title/Abstract] OR "criterion validity"[Title/Abstract] OR "test-retest reliability"[Title/Abstract] OR "item analysis"[Title/Abstract] OR "measurement properties"[Title/Abstract] OR "measurement invariance"[Title/Abstract]) AND ("Achuar"[Title/Abstract] OR "Aimara"[Title/Abstract] OR "Amahuaca"[Title/Abstract] OR "Arabela"[Title/Abstract] OR "Ashaninka"[Title/Abstract] OR "Asheninka"[Title/Abstract] OR "Awajún"[Title/Abstract] OR "Bora"[Title/Abstract] OR "Cashinahua"[Title/Abstract] OR "Chamicuro"[Title/Abstract] OR "Chapra"[Title/Abstract] OR "Chitonahua"[Title/Abstract] OR "Ese Eja"[Title/Abstract] OR "Harakbut"[Title/Abstract] OR "Ikitu"[Title/Abstract] OR "Iñapari"[Title/Abstract] OR "Iskonawa"[Title/Abstract] OR "Jaqaru"[Title/Abstract] OR "Jíbaro"[Title/Abstract] OR "Kakataibo"[Title/Abstract] OR "Kakinte"[Title/Abstract] OR "Kandozi"[Title/Abstract] OR "Kapanawa"[Title/Abstract] OR "Kichwa"[Title/Abstract] OR "Kukama Kukamiria"[Title/Abstract] OR "Madija"[Title/Abstract] OR "Maijuna"[Title/Abstract] OR "Marinahua"[Title/Abstract] OR "Mashco Piro"[Title/Abstract] OR "Mastanahua"[Title/Abstract] OR "Matsés"[Title/Abstract] OR "Matsigenka"[Title/Abstract] OR "Muniche"[Title/Abstract] OR "Murui-Muinanɨ"[Title/Abstract] OR "Nahua"[Title/Abstract] OR "Nanti"[Title/Abstract] OR "Nomatsigenga"[Title/Abstract] OR "Ocaina"[Title/Abstract] OR "Omagua"[Title/Abstract] OR "Quechua"[Title/Abstract] OR "Resígaro"[Title/Abstract] OR "Secoya"[Title/Abstract] OR "Sharanahua"[Title/Abstract] OR "Shawi"[Title/Abstract] OR "Shipibo-Konibo"[Title/Abstract] OR "Shiwilu"[Title/Abstract] OR "Ticuna"[Title/Abstract] OR "Urarina"[Title/Abstract] OR "Uro"[Title/Abstract] OR "Vacacocha"[Title/Abstract] OR "Wampis"[Title/Abstract] OR "Yagua"[Title/Abstract] OR "Yaminahua"[Title/Abstract] OR "Yanesha"[Title/Abstract] OR "Yine"[Title/Abstract]) Filters: in the last 5 years |

| **DATABASE** | **Search Query** |
| --- | --- |
| LILACS | (("psychometric properties" OR "validity" OR "reliability" OR "internal consistency" OR "factorial structure" OR "construct validity" OR "criterion validity" OR "test-retest reliability" OR "item analysis" OR "measurement properties" OR "measurement invariance"))  AND (("Achuar" OR "Aymara" OR "Amahuaca" OR "Arabela" OR "Ashaninka" OR "Asheninka" OR "Awajún" OR "Bora" OR "Cashinahua" OR "Chamicuro" OR "Chapra" OR "Chitonahua" OR "Ese Eja" OR "Harakbut" OR "Ikitu" OR "Iñapari" OR "Iskonawa" OR "Jaqaru" OR "Jíbaro" OR "Kakataibo" OR "Kakinte" OR "Kandozi" OR "Kapanawa" OR "Kichwa" OR "Kukama Kukamiria" OR "Madija" OR "Maijuna" OR "Marinahua" OR "Mashco Piro" OR "Mastanahua" OR "Matsés" OR "Matsigenka" OR "Muniche" OR "Murui-Muinanɨ" OR "Nahua" OR "Nanti" OR "Nomatsigenga" OR "Ocaina" OR "Omagua" OR "Quechua" OR "Resígaro" OR "Secoya" OR "Sharanahua" OR "Shawi" OR "Shipibo-Konibo" OR "Shiwilu" OR "Ticuna" OR "Urarina" OR "Uro" OR "Vacacocha" OR "Wampis" OR "Yagua" OR "Yaminahua" OR "Yanesha" OR "Yine"))  AND ((peruvian OR peru) OR AB=(peruvian OR peru) OR AK=(peruvian OR Perú)) |
